# Exercise-induced coronary spastic angina with an early repolarization pattern diagnosed after cardiopulmonary resuscitation: a case report

**DOI:** 10.1093/ehjcr/ytaf481

**Published:** 2025-09-22

**Authors:** Tomoari Kuriyama, Kozo Hotta, Kazuto Kujira, Ryoji Taniguchi, Yukihito Sato

**Affiliations:** Department of Cardiology, Hyogo Prefectural Amagasaki General Medical Center, 2-17-77 Higashinaniwa-cho, Amagasaki 660-8550, Japan; Department of Cardiology, Hyogo Prefectural Amagasaki General Medical Center, 2-17-77 Higashinaniwa-cho, Amagasaki 660-8550, Japan; Department of Cardiology, Hyogo Prefectural Amagasaki General Medical Center, 2-17-77 Higashinaniwa-cho, Amagasaki 660-8550, Japan; Department of Cardiology, Hyogo Prefectural Amagasaki General Medical Center, 2-17-77 Higashinaniwa-cho, Amagasaki 660-8550, Japan; Department of Cardiology, Hyogo Prefectural Amagasaki General Medical Center, 2-17-77 Higashinaniwa-cho, Amagasaki 660-8550, Japan

**Keywords:** Case report, Exercise-induced coronary spastic angina, Cardiopulmonary exercise test, Early repolarization pattern

## Abstract

**Background:**

Early repolarization pattern is known to be an independent risk factor of ventricular fibrillation in patients with coronary spastic angina. Additionally, drug provocation tests for coronary vasospasms are highly diagnostic, but negative results cannot completely rule out the presence of coronary spastic angina. We report a rare case of cardiopulmonary arrest with early repolarization pattern in a patient diagnosed with cardiopulmonary exercise testing-induced coronary spastic angina.

**Case summary:**

A 36-year-old male suffered a sudden cardiac arrest during a football game. After resuscitation, a normal electrolyte balance was confirmed, and both coronary and head computed tomography scans revealed no abnormalities. Sodium channel blocker and electrophysiological tests were negative. The electrocardiogram revealed early repolarization pattern as the only positive finding. After the examination, we implanted a subcutaneous implantable cardioverter defibrillator for secondary prevention. During cardiopulmonary exercise testing to evaluate his exercise tolerance, the patient experienced sudden chest pain and ST elevation on lead aVR when he reached the anaerobic threshold. He had a poor increase in the oxygen consumption/heart rate. This suggested a decreased cardiac output due to an acute ischaemic attack. The patient was diagnosed with exercise-induced coronary spastic angina. After smoking cessation and drug treatment initiation, no ischaemic symptoms appeared at the anaerobic threshold during cardiopulmonary exercise, and his cardiac output improved. The subcutaneous implantable cardioverter defibrillator never needed to deliver any therapies.

**Discussion:**

This case demonstrated the value of cardiopulmonary exercise testing in diagnosing exercise-induced coronary spasms and its effectiveness in determining the treatment efficacy.

Learning points

- In cases of cardiopulmonary arrest with early repolarization pattern accompanied by diurnal variation, ruling out coronary spastic angina is important.
- Even if the acetylcholine test is negative, exercise-induced coronary spastic angina may be diagnosed by applying an exercise load that reaches the anaerobic metabolic threshold.
- Cardiopulmonary exercise testing may be useful for diagnosing exercise-induced coronary spastic angina and evaluating treatment outcomes.

## Introduction

Early repolarization patterns (ERPs) are well-known electrocardiographic patterns associated with idiopathic ventricular fibrillation (VF), and it has been suggested that proarrhythmic factors such as myocardial ischaemia may be involved in the development of VF.^1^ In addition, ERPs have been reported in patients with coronary spastic angina (CSA) complicated with VF. In particular, CSA cases with a circadian-type ERP are considered high risk for VF recurrence.^2^ Among CSA cases, exercise-induced CSA is rare and difficult to diagnose.^3^ Herein, we report a case of cardiopulmonary arrest with an ERP that led to the diagnosis of exercise-induced CSA during cardiopulmonary exercise (CPX) testing, despite a negative acetylcholine spasm test.

## Summary figure

**Figure:**
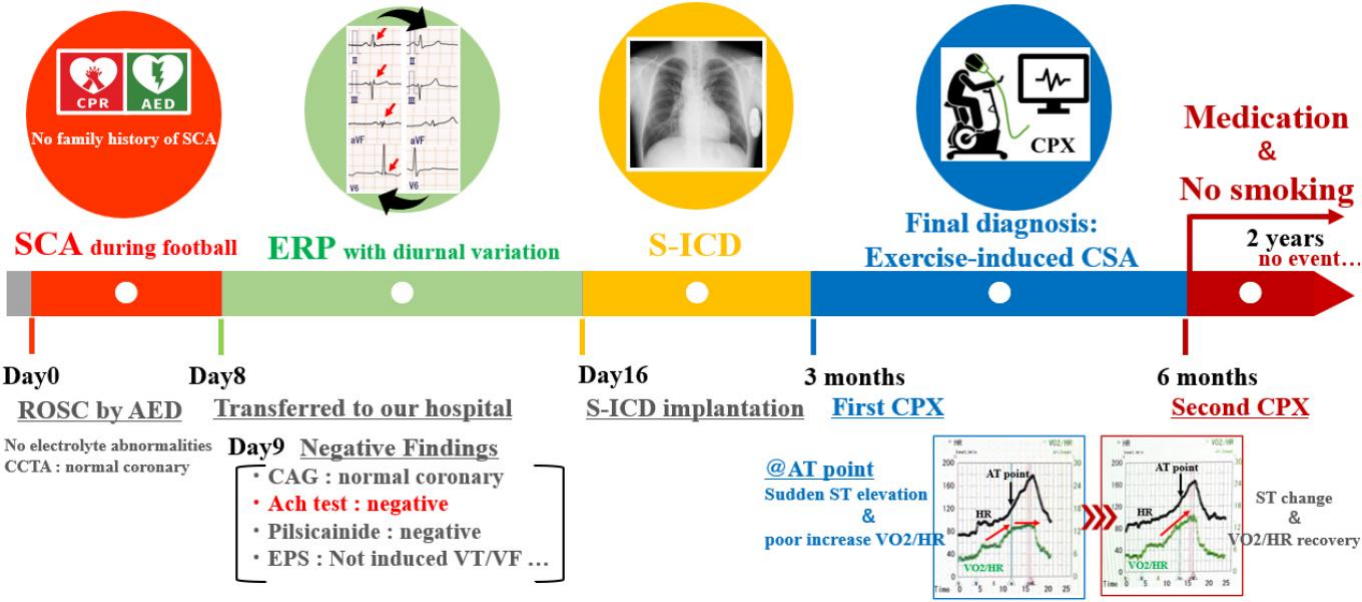


## Case presentation

**Figure 1:**
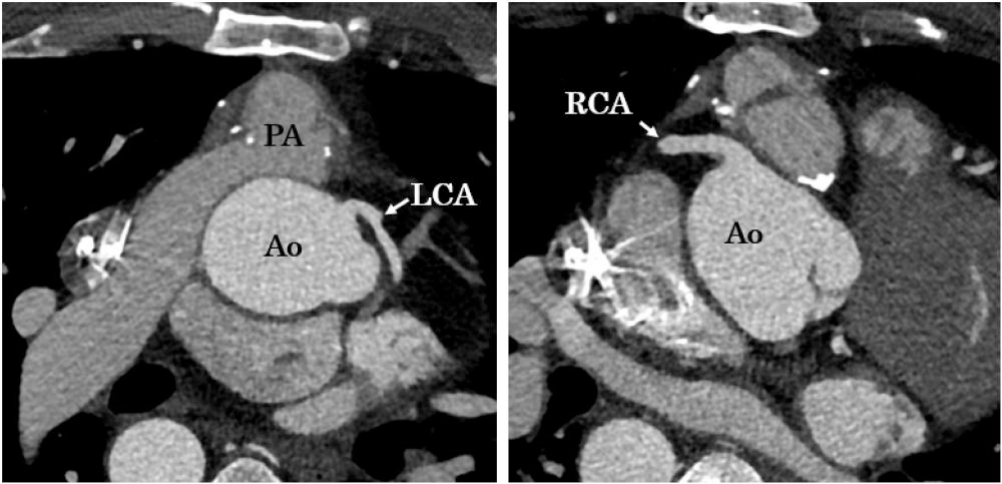
Contrast-enhanced CT scan. No coronary artery stenosis, occlusion, or pulmonary artery stenosis was shown. CT, computed tomography; LCA, left coronary artery; RCA, right coronary artery; Ao, aorta; PA, pulmonary artery.

**Figure 2:**
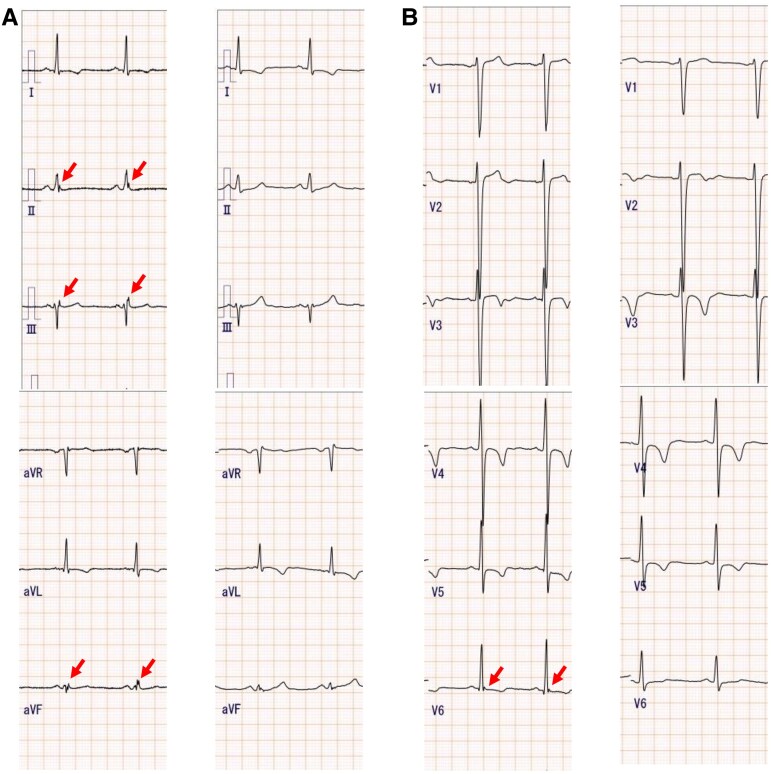
Twelve-lead electrocardiogram. (*A*) An ERP was indicated on the inferior wall and in the V6 lead. (*B*) The ERP disappeared with a diurnal variation. ERP, early repolarization pattern.

**Figure 3:**
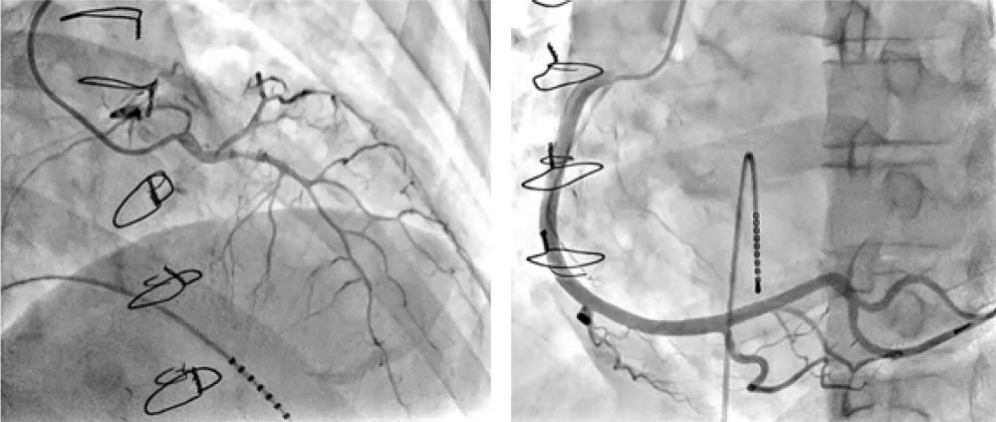
Transcatheter coronary angiography. ACh was injected into the LCA at doses of 20, 50, and 100 µg and into the RCA at doses of 20 and 50 µg. During the ACh test, no ST-T changes on the electrocardiogram or chest symptoms occur (left, left coronary artery after the administration of 100 µg; right, right coronary artery after the administration of 50 µg). ACh, acetylcholine hydrochloride; LCA, left coronary artery; RCA, right coronary artery.

**Figure 4:**
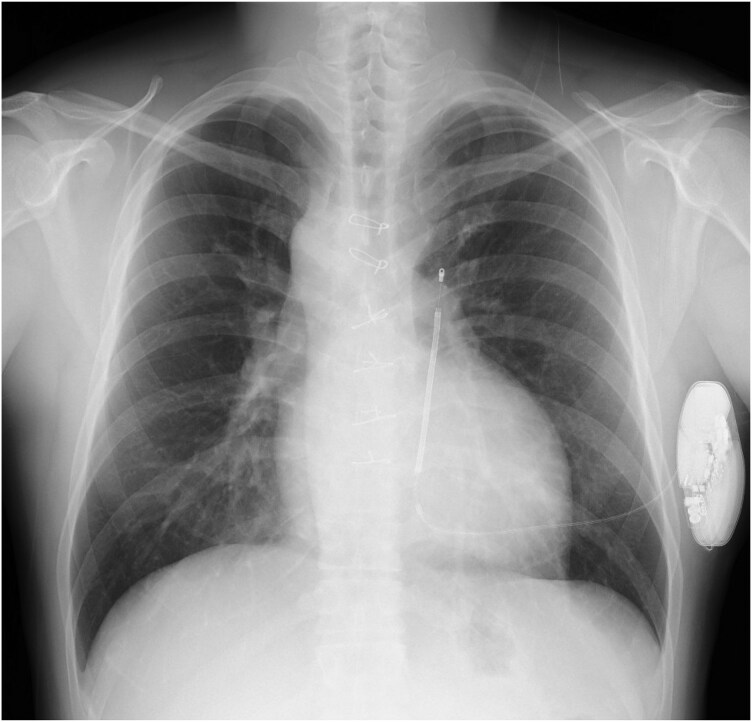
X-ray of the S-ICD. S-ICD, subcutaneous implantable cardioverter defibrillator.

A 36-year-old man, who underwent a Jatene operation at the age of 3 months for a complete transposition of the great arteries, experienced sudden cardiopulmonary arrest during a football game. He also fainted during football 4 years prior and was under observation without an examination. After resuscitation, he was taken to a hospital by ambulance. No electrolyte abnormalities were detected. The echocardiogram showed no abnormal findings in the cardiac function and no progression of pulmonary artery stenosis. Computed tomography also indicated no obvious coronary or pulmonary artery stenosis (*Figure 1*). On Day 8, the patient subsequently visited our hospital and underwent implantation of an implantable cardioverter defibrillator (ICD). Electrocardiography revealed an ERP with a diurnal variation on the inferior wall and in lead V6 (*Figure 2*). On Day 9, we performed coronary angiography. No coronary stenosis was shown. Acetylcholine spasm testing was performed, and spasms were provoked with intracoronary injections of 20 and 50 µg of acetylcholine into the right coronary artery and 20, 50, and 100 µg into the left coronary artery as recommended by the Circulation Society guidelines.^4^ We could not confirm any transient luminal narrowing of >90%, ischaemic electrocardiogram findings, or chest symptoms (*Figure 3*). A pilsicainide stress test was negative. Given that the sinus rate was 100 b.p.m./min at the initiation of the electrophysiology test, isoprenaline was not administered. Instead, programmed ventricular stimulation was performed with triple extrastimuli at two different right ventricular sites,^5^ but no ventricular arrhythmias were induced. Therefore, we implanted a subcutaneous ICD (S-ICD) on day 16 (*Figure 4*). In addition, he had no family history of sudden cardiac death and declined genetic testing.

**Figure 5:**
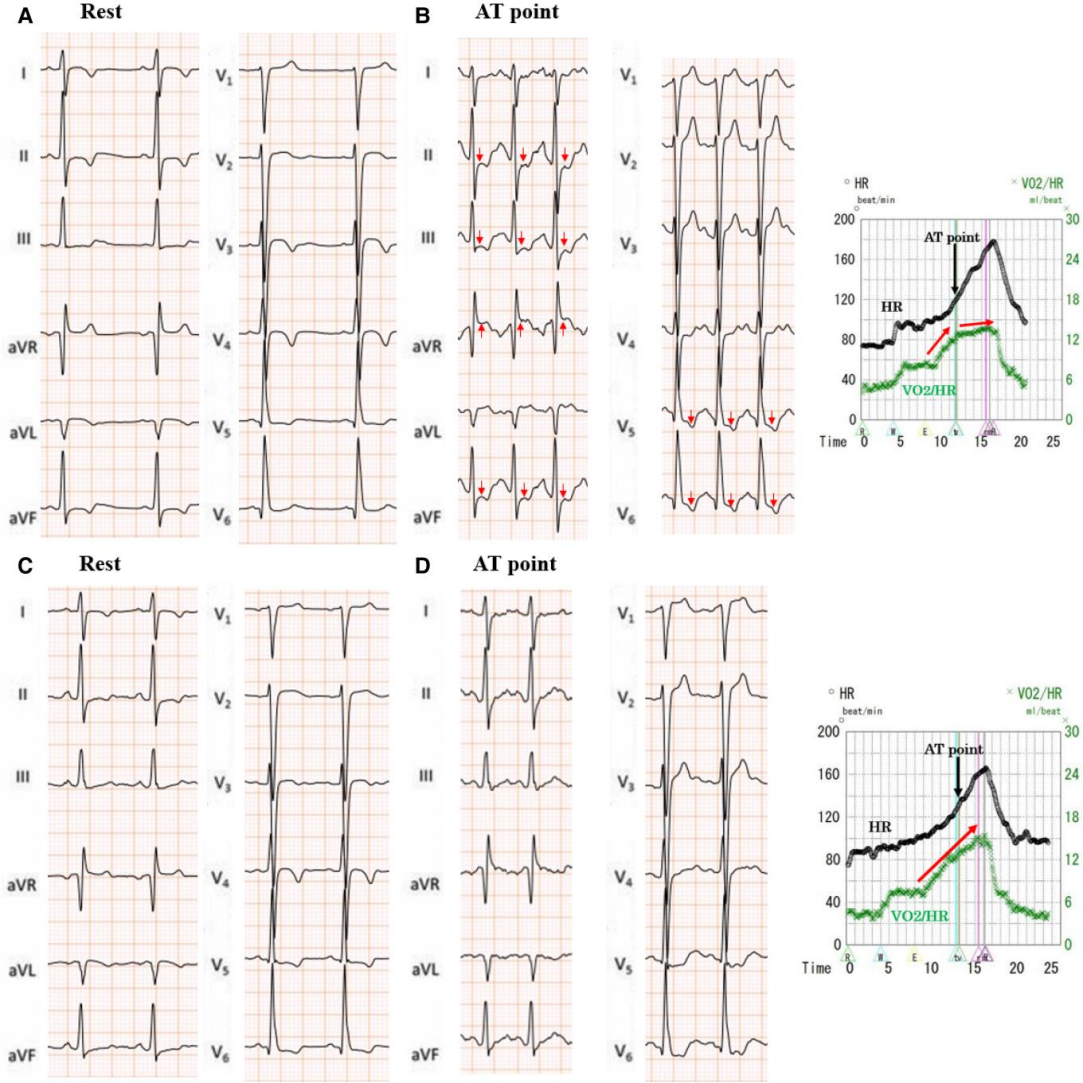
Cardiopulmonary exercise test. The test was performed on a bicycle ergometer, using the 20 W/min ramp protocol. (*A*) First round of CPX treatment: on reaching the AT at 180 W, the patient suddenly experienced chest discomfort, and the electrocardiogram exhibited ST elevation in lead aVR and ST depression in the inferior wall and V4–V6 leads. (*B*) When the AT was reached, he had a poor increase in the VO_2_/HR, indicating a sudden decrease in the cardiac output associated with an acute ischaemic attack. (*C*) Compared with the initial ECG, the post-treatment CPX levels indicated that the ischaemic changes caused by the coronary spasms had improved. (*D*) Even after reaching the symptomatic limit, the VO_2_/HR increased in one direction, indicating that the cardiac output was maintained. CPX, cardiopulmonary exercise test; ECG, electrocardiogram; VO_2_, oxygen consumption; HR, heart rate; AT, anaerobic threshold.

Cardiopulmonary exercise testing was performed to assess his exercise tolerance 3 months after discharge. Before starting the CPX, the acceptability of the electrocardiograms, blood pressure, and expired gas data at rest was checked. The CPX began after 4 min of rest. After a 4 min, 20 W warm-up, a symptom-limited exercise test was performed using a 20 W/min ramp protocol on a cycle ergometer. Although the peak VO_2_ was 30.4 mL/min/kg (104%) and exercise tolerance was good, the anaerobic threshold was reached at 180 W. However, the patient experienced shortness of breath, and thought it was because of the exercise load, but suddenly a significant ST elevation was observed in the aVR lead with ST depression on the inferior wall and V4–V6 leads of the ECG (*Figure 5A*). Although the heart rate (HR) was elevated, the VO_2_/HR decreased, suggesting a decreased cardiac output (*Figure 5B*). The examination was immediately terminated. The symptoms improved with the administration of nitrates, and the electrocardiogram normalized. Therefore, we diagnosed him with exercise-induced CSA.

We instructed the patient to stop smoking and initiated diltiazem (200 mg daily) and nicorandil (15 mg daily) treatment. When subsequent CPX testing was performed 5 months later, no ischaemic symptoms occurred, and his exercise tolerance improved significantly (*Figure 5C* and *D*). We have followed up the patient for 2 years. With ongoing remote monitoring using the S-ICD, only one non-sustained ventricular tachycardia lasting 5 s was observed. There has been no fatal arrhythmia.

## Discussion

Two lessons were learned from this case. Firstly, we should suspect exercise-induced CSA in cases with cardiac arrest upon exercise, particularly in those exhibiting an ERP with a diurnal variation, even if pharmacologic stress tests are negative. Secondly, CPX testing may be effective in diagnosing exercise-induced CSA and evaluating the effects of the treatment.

The sensitivity and specificity of acetylcholine tests in patients with CSA are more than 90%, with a high diagnostic performance, and morning testing is known to increase inducibility.^6^ On the other hand, previous reports have shown that a positive rate of acetylcholine tests in patients with CSA is significantly lower in those under 40 years old than in older patients (33.3% vs. 71.5%; *P* < 0.05).^6^ There have also been reports documenting symptomatic CSA with ST-segment elevation on exercise testing despite a negative acetylcholine stress test.^7,8^ Recent cases have been reported regarding the use of handgrips for the diagnosis of exercise-induced CSA, suggesting the simplicity, usefulness, and safety of that test during loading.^9^ However, in the present case, rapid electrocardiogram changes and a decreased cardiac output appeared as soon as the symptomatic limit was reached, and it was difficult to determine whether the breathlessness was due to ischaemic symptoms or not. The previous syncope on exertion episodes may also have been cardiogenic. In other words, in the present case, high-intensity exercise to reach the anaerobic threshold may have been necessary to induce the coronary spasms.

In this situation, an ERP may provide insight into the presence of potentially severe coronary angina; Kitamura *et al*.^2^ reported that an ERP was a VF independent factor in patients with CSA, with a positive rate of 61.9%. They also found that when ERPs exhibited diurnal fluctuations, the rate of VF recurrence also increased, but the pathological relevance was not clear.^2^ Wang *et al*.^10^ also found that CSA cases with ERPs on the inferior wall had an increased risk of VF occurrence during coronary spasms (OR = 7.80; 95% CI, 4.04 15.05; *P* < 0.001). Therefore, this case had a high risk of VF complications, even for a CSA patient, and the risk of recurrence must also be considered. Further, the patient had a history of a Jatene surgery that corrected a congenital heart defect where the aorta and pulmonary artery were switched. The surgeon detached and reattached the aorta and pulmonary artery to the opposite ventricles. One of the long-term complications associated with the Jatene procedure is coronary artery stenosis.^10^ Furthermore, myocardial bridging can cause CSA in patients with a complete transposition of the great arteries,^11,12^ but we could not show this in our patient. It will be necessary to carefully monitor his postoperative course.

Cardiopulmonary exercise testing may be diagnostically useful in cases of cardiopulmonary arrest in young people with high activity levels, especially in cases occurring during competitive sports. In this case, CPX testing was useful for accurately evaluating the efficacy of pharmacological therapy and smoking cessation therapy by monitoring the electrocardiogram changes and cardiac output. However, previous reports indicated that the incidence of VF during exercise-induced testing in CSA patients was 1.8%.^13^ Therefore, when performing CPX testing, it is important to have emergency response preparations in place, such as ICDs and medications. Even if the diagnosis of CSA was reached before the ICD was implanted, Matsue *et al*.^14^ reported that patients with CSA who had a fatal arrhythmia were at high risk of a recurrent cardiac arrest even if they did not feel chest pain with appropriate drug treatment, so it was thought that ICD was implanted to prevent sudden death.

In this case report, we presented a case of exercise-induced CSA with an ERP accompanied by a diurnal variation, which was associated with a high risk of VF recurrence and a diagnosis using CPX testing. Performing exercise stress testing in cases of unexplained cardiopulmonary arrest during exercise may be critical for the detection of high-risk CSA.

## Lead author biography


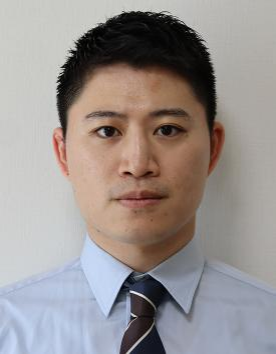


Tomoari Kuriyama graduated from Fukui University in 2016 and has been working as a cardiologist in Hyogo Prefectural Amagasaki General Medical Center since 2021. He specialized in clinical and research imaging fields such as electrophysiology and heart failure.

## Data Availability

The data underlying this article will be shared on reasonable request to the corresponding author.
